# On Strangulated Hernia, and the Use of Chloroform for Its Reduction

**Published:** 1849-06

**Authors:** Michel Guyton


### SURGERY.

*On Strangulated Hernia, and the use of Chloroform for its Reduction.* By M. Michel Guyton.—The author, after stating the usual causes of strangulation, points out that the distension by gas is one of the most frequent causes of irreducibility. Irreducibility, in consequence of an accumulation of gas, is the first symptom of strangulated hernia. It is simply a gaseous tumour, which, from purely mechanical laws, comes to be tightly impacted in an opening through which it has passed; but soon the vitality of its coats comes into play, and inflammation and other secondary lesions are the natural and inevitable consequences, and these are what give hernia its dangerous and justly formidable characters. At first, reduction by ordinary taxis is possible, and generally will be easily accomplished; it depends on the force of contraction in the abdominal muscles, the degree of distension of the hernia, and, above all, on the width of the opening of communication. If one cannot always reduce it, it is owing to the difficulty of exercising on the intestine a pressure sufficient to counterbalance that of the whole abdomen: that is to say, because our force is employed upon a small extent of surface, and one difficult to compress. With chloroform, one has not to combat muscular action, the gases are quickly dispersed, and the intestinal walls collapse and readily re-enter. Further, distension of strangulated hernia by gas, permanent and involuntary contraction of the abdomen, constitute the two principal elements of strangulation; pain is only secondary—its importance lies entirely in its results, namely, the muscular contraction. Chloroform acts only on this contraction and on pain; the rings remain in the same condition—they cannot be slackened either by abolition of muscular force, which has no effect on them, nor yet by direct relaxation. Chloroform, in destroying the true and principal cause of strangulation, namely, contraction, destroys also its results: the hernia empties itself, and is reduced. It follows, therefore, that chloroform is most called for in hernia composed entirely of intestine; and that the reduction of compound hernia is more difficult than that of simple enterocele, nay even at times impossible. Chloroform might be useful in those cases of epiplocele, and entero-epliplocele where omentum predominates, by abolishing the pain produced by the various manœuvres, and that involuntary and powerful contraction of the muscles by which every organ in the abdomen has a tendency to escape. But it is no longer so direct in its influence; the omentum forms without the ring a hard solid tumour, incapable of being reduced suddenly by pressure, and, if its diameter is very much disproportioned to that of the opening, its return is quite impossible.—*London Monthly Journal from L'Union Med.*

